# popharvest: An *R* package to assess the sustainability of harvesting regimes of bird populations

**DOI:** 10.1002/ece3.8212

**Published:** 2021-11-15

**Authors:** Cyril Eraud, Tiphaine Devaux, Alexandre Villers, Fred A. Johnson, Charlotte Francesiaz

**Affiliations:** ^1^ Office Français de la Biodiversité Direction de la Recherche et de l'Appui Scientifique Unité Avifaune migratrice Villiers‐en‐Bois France; ^2^ Office de Génie Ecologique Agence Nord‐Est Strasbourg France; ^3^ Centre d'Ecologie et des Sciences de la Conservation – UMR 7204 MNHN‐CNRS‐UPMC Muséum National d'Histoire Naturelle Paris France; ^4^ Aarhus University Department of Bioscience—Kalø Rønde Denmark; ^5^ Office Français de la Biodiversité Direction de la Recherche et de l'Appui Scientifique Unité Avifaune migratrice Ile‐D'Olonne France

**Keywords:** demographic invariant method, harvest, hunting management, potential take level, sustainability

## Abstract

Bird harvest for recreational purposes or as a source for food is an important activity worldwide. Assessing or mitigating the impact of these additional sources of mortality on bird populations is therefore crucial issue. The sustainability of harvest levels is however rarely documented, because knowledge of their population dynamics remains rudimentary for many bird species. Some helpful approaches using limited demographic data can be used to provide initial assessment of the sustainable use of harvested bird populations, and help adjusting harvest levels accordingly. The *Demographic Invariant Method* (DIM) is used to detect overharvesting. In complement, the *Potential Take Level* (PTL) approach may allow setting a level of take with regard to management objectives and/or to assess whether current harvest levels meet these objectives. Here, we present the R package popharvest that implements these two approaches in a simple and straightforward way. The package provides users with a set of flexible functions whose arguments can be adapted to existing knowledge about population dynamics. Also, popharvest enables users to test scenarios or propagate uncertainty in demographic parameters to the assessment of sustainability through easily programming Monte Carlo simulations. The simplicity of the package makes it a useful toolbox for wildlife managers or policymakers. This paper provides them with backgrounds about the DIM and PTL approaches and illustrates the use of popharvest's functionalities in this context.

## Graphical abstract

The paper introduces a didactic overview of two approaches that can be used to assess the sustainability of harvest regimes of birds when demographic knowledge is limited. These approaches are implemented in a new R package providing wildlife managers with a useful toolbox for prioritizing research works or management actions.

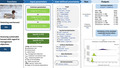

## 1 INTRODUCTION

Addressing the demographic consequences of additional—human‐induced—sources of mortality on bird populations is a central topic in conservation biology. Both sedentary and migratory birds face a large array of additional sources of mortality to natural ones, with types and importance varying according to economical or cultural contexts (Drewitt & Langston, 2008; Eason et al., 2016; Hirschfeld & Attard, 2017; Loss et al., 2015; Munilla et al., 2011; Yeh et al., 2013).

Hunting for recreational purposes is one of the major sources of additional mortality. A large number of species are legally exposed to various harvest regimes at continent scales, with millions of birds harvested each year (e.g., Hirschfeld & Attard, 2017). In this context, addressing the demographic consequences of hunting activity is an important issue to assess whether regulatory commitments and underlying conservation objectives are met (e.g., AEWA, 2018; EU Birds Directive, 2009). However, the sustainability of harvest levels is rarely documented which constitutes a source of conflicts between hunters and organizations in charge of conservation.

For numerous species, the lack of detailed knowledge of their full life cycle hinders the ability of assessing harvest sustainability. Demographic information is often limited to coarse estimates for a few parameters (e.g., Dillingham & Fletcher, 2008), and their temporal variance is poorly documented. Detailed information on hunting bags is also limited for most countries. Total harvests by country are often the only existing statistics used to characterize harvest regimes, with no further information about how those estimates were obtained (hence their robustness), and how they balance among sex and/or age‐classes (e.g., Hirschfeld & Attard, 2017). This situation may preclude the use of fine‐tuned matrix population models. However, alternative approaches using sparse/incomplete data can be used to provide initial assessment of the sustainable use of harvested bird populations, and help wildlife managers or policymakers to adjust harvest levels accordingly.

One of these approaches consists in comparing hunting bags with annual growth rate for a population under optimal conditions, that is, at low density and with no limiting factors (Niel & Lebreton, 2005). Under such a situation, population growth rate (*λ*_max_) is maximized and the population increases by a proportion of *λ*_max_–1 each year (i.e., *maximum recruitment rate*, *r*_max_; Dillingham & Fletcher, 2008). Given that such a limitation‐free situation is rarely observed in nature, any harvest level that exceeds this potential excess growth is a cue of overharvesting, which may ultimately lead to population depletion or extinction (Robinson, 2000). Niel and Lebreton (2005) further extended this approach, then solutioning the challenge of estimating *λ*_max_ under optimal or “*rarified*” conditions (Johnson et al., 2012). Their procedure, termed the *Demographic Invariant Method* (DIM), allows estimating *λ*_max_ on the basis of very few demographic parameters such as adult survival and age at first breeding, considering tight and permanent allometric relationships among demographic parameters. DIM is therefore a useful approach to assess whether some additional sources of mortality like hunting are unsustainable, even in poorly studied species. However, the DIM formulation does not include a priori management objectives in its formulation. Consequently, this approach cannot assess whether current harvest levels are sustainable with regard to the objective of maintaining populations at levels complying with some ecological, economical, or cultural requirements.

A complementary approach that can be used for this purpose is the *Prescribed Take Level* method (PTL, Runge et al., 2009). This approach is a generalization of the *Potential Biological Removal* (PBR) initially developed to assess the sustainability of human‐caused mortality in marine mammals (Wade, 1998). The PTL is grounded on the theory of density‐regulated population growth, which can be formulated as a discrete theta‐logistic model allowing for linear or nonlinear density dependence (Johnson et al., 2012). The PTL uses properties of this formulation to derive a maximum sustainable yield (MSY), which, over time, drives the population size at an equilibrium point corresponding to a fraction of the carrying capacity (*K*, Runge et al., 2009). Then, MSY can be used to set a harvest strategy or can be compared to current hunting bags to assess whether they are sustainable with regard to a predefined management objective. In practice, the PTL approach requires few parameters and is therefore a useful starting point for assessing allowable take within a bird population with limited demographic information. These parameters include population size, *r*_max_ (or *λ*_max_–1) and a shape parameter describing the shape of the density‐dependent relationship. Management objective is a priori specified within the formulation of the PTL as a scaling factor representing the desired population size relative to K and the take level relative to MSY (Johnson et al., 2012; Koneff et al., 2017; Runge et al., 2009).

In data‐poor environments, the DIM and PTL approaches represent two facets of how to detect overharvest and setting allowable take of birds that may satisfy some management or conservation objectives (e.g., Johnson et al., 2012; Koneff et al., 2017; Lormée et al., 2020; Turrin & Watts, 2016; Watts et al., 2015). However, computer tools that implement DIM and PTL algorithms in a flexible and accessible way to a broad audience are lacking. Here, we introduce a user‐friendly R package popharvest, which allows the conduct of such assessments within an open‐source interface and in a flexible *R* programming language.

## 2 PACKAGE OVERVIEW

The R package popharvest implements the DIM and PTL approaches through a set of highly flexible functions whose arguments can be adapted to existing knowledge on the population dynamics, including situations where demographic data are very limited. In particular, the package makes available to users a series of functionalities derived from the work of Johnson et al. (2012), which can be easily mobilized when some of the key parameters used for the calculations (e.g., survival rate, shape of the density dependence) are virtually unknown. popharvest also provides users with a powerful tool for explicitly incorporating uncertainty/stochasticity in calculations, which may help craft more realistic management decisions or may help testing scenarios. This is done by substituting point estimates of demographic parameters by probability distributions (e.g., log‐normal, uniform) and performing Monte Carlo simulations by the means of an easy programming syntax. Functions for visualizing, calculating various statistics, and exporting results are also implemented.

## 3 METHODS AND IMPLEMENTATION

### 3.1 Detecting overharvest: the DIM approach

Under optimal conditions, a population increases by an annual proportion equals to *r*_max_ (or *λ*_max_−1). Accordingly, any level of mortality that exceeds this potential excess growth (i.e., Mortality/Potential excess growth >1) is unsustainable. However, we stress that the inverse is not true: Any mortality levels below potential excess growth are not synonymous of sustainability (see Niel & Lebreton, 2005; Robinson, 2000). The function PEG provides users with an algorithm that performs the assessment on the basis of few demographic information.

The potential excess growth (PEG) is calculated following Niel and Lebreton (2005):

(1) $$\text{PEG}=N\times \left(\lambda_{\max}‐1\right)\times Fs$$

where *N* is the population size and *λ*_max_ is the maximal annual growth rate measured under optimal conditions (i.e., at low density and with no limiting factors). *Fs* is a «safety» parameter taking values within the [0–1] range, which allows users taking only a proportion of the maximal PEG when performing their assessments of sustainability. Several arguments are given in Niel and Lebreton (2005) justifying its use to reduce the risk of drawing wrong conclusions. For instance, a species or population may be exposed to other unquantified sources of additional mortality, which may accumulate to value above maximum PEG. Current harvests may also be imbalanced toward age‐classes with the highest reproductive value, then increasing the potential for negative demographic consequences. In addition, populations exposed to harvest can be small and/or subject to high environmental stochasticity, then needing a conservative approach. As a rule, *Fs* needs to be a priori defined and should not exceeds 0.5 (Niel & Lebreton, 2005; Wade, 1998). One optional approach is to set *Fs* according to species’ IUCN conservation status (Dillingham & Fletcher, 2008): 0.5 for «Least concern (LC)» species, 0.3 for «Near threatened (NT)» and 0.1 when they are categorized as «Vulnerable (VU)».

Having *N*, *λ*_max_, the mortality level and setting *Fs*, the user supplies the corresponding values to arguments listed in the overharvest function (respectively: pop.fixed, lambdaMax, harvest.fixed, Fs). The user can specify up to 3 values for *Fs*, which allows estimations to be made for different management/conservation scenarios for one species/population (NSp=1). The function output.summary() returns a *sustainable harvest index* (SHI) calculated as the ratio Mortality/Potential excess growth. A value >1 suggests unsustainability.



PEG(pop.fixed=10000,NSp=1,Fs=c(0.1,0.2,0.3),lambdaMax.fixed=1.35, harvest.fixed=1500)




In most cases, *λ*_max_–1 (or *r*_max_) is an unknown quantity as populations face limiting factors and consequently grow under nonoptimal conditions. Niel and Lebreton (2005) hence developed a procedure to estimate *λ*_max_, taking advantage of the relationship linking *λ*_max_ to (optimal) generation time. Under this procedure, *λ*_max_ is derived from estimates of the maximal (annual) adult survival (*S*_a_) and the age at first reproduction (*α*, in year). The function PEG allows users to estimate *λ*_max_ using this approach, by simply replacing the argument lambdaMax by the arguments surv. (*S*_a_) and alpha. (*α*). One subtlety of the procedure is that it needs to be adapted to the species’ life‐history strategy (Niel & Lebreton, 2005).

For long‐lived species, *λ*_max_ is estimated by solving:

(2) $$\lambda_{\max}\approx \frac{\left(s\alpha ‐s+\alpha +1\right)+\sqrt{{\left(s‐s\alpha ‐\alpha ‐1\right)}^{2}‐4s\alpha^{2}}}{2\alpha }$$

For short‐lived species, *λ*_max_ is estimated by numerically solving:

(3) $$\lambda_{\max}=\exp\left[{\left(\alpha +\frac{s}{\lambda_{\max}‐s}\right)}^{‐1}\right]$$

The argument living.rate="short" or "long" is then passed to the function to call one of these two formulas.



PEG(pop.fixed=10000, NSp=1, Fs=0.3, surv.fixed=0.80, alpha.fixed=1, living.rate="short", harvest.fixed =1500)




Calculations for *n* species or populations can simultaneously be performed in a single run. The argument NSp=*n* is passed to the function, and the values of the function arguments are then filled as a vector of length *n* in which their order follows the sequence of the *n* studied populations/species.



PEG(NSp=2, living.rate = c("long", "short"), surv.fixed = c(0.8, 0.65), alpha.unif = TRUE, min.alpha = c(2, 1), max.alpha = c(2, 1), pop.fixed = c(3600000, 55000000), harvest.fixed = c(100000, 8000000), Fs = c(0.1, 0.3, 0.5))




One of the assumptions of the DIM approach is that survival estimates correspond to the maximum values (*S*_a_) measured under optimal conditions. If *S*_a_ is underestimated, this increases the resulting *λ*_max_, and consequently, the PEG value used as a comparison threshold with current harvest levels. Banding and capture–recapture designs are common tools to assess survival in free‐ranging birds, but careful consideration must be given to their accuracy. For instance, emigration may negatively bias survival estimates derived from live‐recaptures (Ergon & Gardner, 2014). Some approaches may overcome this problem, such as spatial capture–recapture (Ergon & Gardner, 2014) or dead recovery models fitted at large geographic scales (Brownie et al., 1985). However, insofar as the species or population is exposed to harvest, the observed survival may be lower than the true survival achieved in the absence of this additional source of mortality. Using the upper interval bounds of estimates of adult survival may be an option. However, there are situations where no estimates of survival are available or, alternatively, those existing are not useful because they rely upon obsolete or inappropriate sampling designs/methodologies (e.g., life tables in old 1960s publications).

One solution to this problem is that, in birds, *S_a_* can be estimated from an allometric relationship linking maximal adult survival and species body mass. This relationship was formulated by Johnson et al. (2012) on the basis of survivorship data measured on captive birds (Ricklefs, 2000):

(4) $$S_{a}={\left(\frac{p}{S_{j}}\right)}^{1/(\exp\left[3.22+0.24\times \ln\left(M\right)+e\right]‐\alpha )}$$

*S_j_* is (average) annual survival for birds aged 1 year to *α*, and *p* is defined from a beta distribution ~*β*(3.34, 101.24). *M* is body mass (in kg), *e* are normally distributed residuals ~*N*(0, *σ*² = 0.087), and *α* is age at first breeding (in years). This solution is implemented in the PEG function. To do that, the user replaces the argument related to survival (surv.fixed) by mass.fixed. With the arguments type.p and type.e="determinist", *p* and *e* are set at their means (i.e., 3.34/(3.34+101.24) and 0). When type.p and type.e="random" are used, *p* and *e* are sampled within their respective distributions (Table 1). α is specified either as a fixed value (alpha.fixed) or drawn from a uniform (alpha.unif) or a log‐normal (alpha.lognorm) distribution. By default, the *S_j_* argument is silent (i.e., *S_j_* = 1), assuming that the situation *S_j_* < *S*_a_ does not hold for birds aged 1 to *α*. In contrary case, the user can provide a value for *S_j_* by using the (fixed) argument surv.j. Full details on estimating survival from body mass are provided in Appendix 1.

**TABLE 1:** List and description of arguments for the PEG^(1)^ and PTL^(2)^ functions

Arguments	Argument types	Description	Examples
Pop.fixed^(1,2)^	Integer	Point estimate of population size	Pop.fixed=10000
pop.unif^(1,2)^	Boolean (Default = FALSE)	Calls the function to draw population size from a uniform distribution bounded with minimum (min.pop) and maximum (max.pop) values	pop.unif=TRUE, min.pop=6000, max.pop=12500
pop.lognorm^(1,2)^	Boolean (Default = FALSE)	Calls the function to draw population size from a log‐normal distribution ln *N*(mean.pop, sd.pop)	pop.lognorm=TRUE, mean.pop=6000, sd.pop=130
Rmax.fixed^(1,2)^	Decimal	Point estimate of maximal annual recruitment rate	Rmax.fixed=0.79
Rmax.lognorm^(1,2)^	Boolean (Default = FALSE)	Calls the function to draw *R*_max_ from a log‐normal distribution ln *N*(mean. *R*_max_, sd. *R*_max_)	Rmax.lognorm=TRUE, mean. Rmax=XX, sd. Rmax=XX
lambdaMax^(1,2)^	Decimal	Point estimate of maximal annual growth rate	lambdaMax=1.35
lambdaMax.lognorm^(1,2)^	Boolean (Default = FALSE)	Calls the function to draw lambda max from a log‐normal distribution ln *N*(mean. lambdaMax, sd. lambdaMax)	lambdaMax .lognorm=TRUE, mean. lambdaMax =6000, sd. lambdaMax =130
surv.fixed^(1,2)^	Decimal (range 0–1)	Point estimate of adult annual survival	surv.fixed=0.80
surv. beta^(1,2)^	Boolean (Default = FALSE)	Calls the function to draw survival from a beta distribution. The function uses the method of moments to specify associated parameters from mean and sd.	surv. beta=TRUE, mean. surv =0.80, sd. surv =0.12
surv.j^(1,2)^	Decimal (range 0–1)	Point estimate of the (average) annual survival (*S_j_*) for birds aged 1 to *α* (age at first breeding). Argument used when estimating adult survival from body mass (i.e., to calculate *p*/*S_j_*) in situation when *S_j_* < *S_a_* (adult survival) for birds aged 1 to *α*. Default is silent, assuming *S_j_* < *S_a_* solely for birds aged <1 year.	surv.j = 0.66
alpha.fixed^(1,2)^	Decimal	Point estimate for the age at first breeding	alpha.fixed=1
alpha.unif^(1,2)^	Boolean (Default = FALSE)	Calls the function to draw age at first breeding from a uniform distribution bounded with minimum (min.alpha) and maximum (max.alpha) values	alpha.unif=TRUE, min.alpha=1, max.alpha=2
alpha.lognorm^(1,2)^	Boolean (Default = FALSE)	Calls the function to draw age at first breeding from a log‐normal distribution ln *N*(mean.alpha, sd.alpha)	alpha.lognorm=TRUE, mean.alpha=1.2, sd.alpha=0.3
mass.fixed^(1,2)^	Boolean (Default = FALSE)	Calls the function to estimate adult survival from point estimate of body mass (in kg)	mass.fixed=0.174
mass.lognorm^(1,2)^	Boolean (Default = FALSE)	Calls the function to estimate adult survival from estimates of body mass (in kg) drawn from a log‐normal distribution ln *N*(mean.weight, sd.weight)	mass.lognorm=TRUE, mean.mass=0.174, sd.mass=0.018
type.p^(1,2)^	Character string	Can either be “determinist” or “random.” When it is “determinist,” calls the function to estimate adult survival from body mass while keeping parameter *p* equal to 3.34/(3.34 + 101.24). When it is “random,” calls the function to estimate adult survival from body mass while sampling the parameter *p* of the underlying relationship within the beta distribution *β*(3.34, 101.24)	type.p = “determinist”
type.e^(1,2)^	Character string	Can either be “determinist” or “random.” When it is “determinist,” calls the function to estimate adult survival from body mass while keeping residuals of the underlying relationship at their means (i.e., 0). When it is “random,” calls the function to estimate adult survival from body mass while sampling residuals of the underlying relationship within the normal distribution *N*(0, 0.087)	type.e = “determinist”
harvest.fixed^(1,2)^	Integer	Point estimate of take (mortality) level	harvest.fixed=1500
harvest.unif^(1,2)^	Boolean (Default =FALSE)	Calls the function to draw take level from a uniform distribution bounded with minimum (min.harvest) and maximum (max.harvest) values	harvest.unif=TRUE, min.harvest=1000, max.harvest=2000
harvest.lognorm^(1,2)^	Boolean (Default = FALSE)	Calls the function to draw estimates of take level from a log‐normal distribution ln *N*(mean.harvest, sd.harvest)	harvest.lognorm=TRUE, mean.harvest=1500, sd.harvest=250
theta.fixed^(2)^	Decimal	Point estimate of the shape parameter describing the shape of the density‐dependent function	theta.fixed=1
estim.theta^(2)^	Character string	Can either be “determinist” or “random.” When it is “determinist,” calls the function to estimate theta from *r*_max_ while keeping residuals of the underlying relationship at their means (i.e., 0). When it is “random,” calls the function to estimate theta from *r*_max_ while sampling residuals of the underlying relationship within the normal distribution *N*(0, 0.942)	estim.theta = “determinist”
full.option	Boolean (Default = FALSE)	If TRUE, provides all parameter values used in calculations.	full.option=TRUE



PEG(pop.fixed=10000, NSp= 1, Fs=0.3, mass.fixed=0.125, type.p = "random", type.e = "random", alpha.fixed=1, living.rate="short", harvest.fixed = 1500)




**FIGURE 1:**
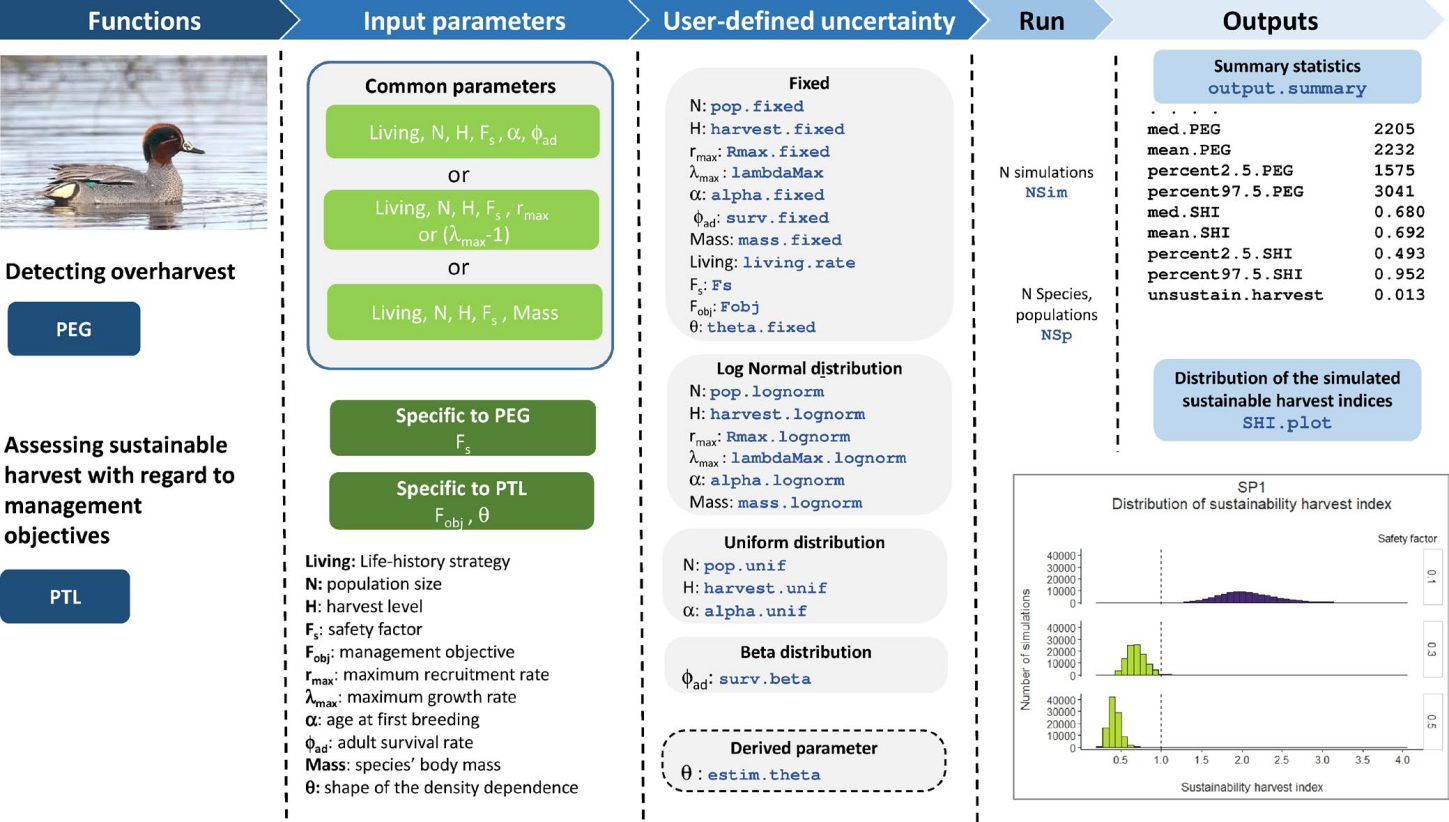
Workflow, functionalities, and main arguments of the package popharvest for assessing the sustainability of harvesting regimes of bird populations. (a) Input parameters are filled by users. The functions accommodate for a priori knowledges of population dynamics and allow for some missing parameters (*r*_max_, *λ*_max_, *ϕ*, *θ*) to be estimated. Some input parameters are common to PEG and PTL functions, and others are functions specific. (b) Uncertainty for some input parameters is specified by users either by setting fixed values or simulating values drawn within a priori distributions. (c) Results are provided as data frames or histograms of sustainable harvest indices distribution. Arguments listed in this figure are detailed in Table 1



The high flexibility of the PEG function allows users to explicitly incorporate uncertainty in parameter values when performing assessment of the sustainable use of a species/population (Figure 1). Survival can be specified as following a beta distribution *Beta* (*α*, *β*) and all others parameters (i.e., population size, *λ*_max_, age at first reproduction, body mass, harvest level) as following a log‐normal distribution ln *N*(*μ*,*σ*), whose associated parameters are specified by the method of moments. Population size, age at first breeding, and harvest level can also be specified as following continuous uniform distributions *U*(lower, upper; Table 1), since these parameters are commonly published as ranges of values. The Nsim argument is then passed to the function to specify the number of Monte Carlo simulations. At each simulation, the function samples within the distributions specified for each argument and then performs calculations. Each simulation and underlying parameters are stored in a data frame (e.g., df).

df<‐PEG(Nsim=100000,NSp=1,Fs=0.3,pop.unif=TRUE,min.pop=10000, max.pop=20000,surv.beta=TRUE,mean.surv=0.65,sd.surv=0.08,alpha.lognorm=TRUE,mean.alpha=1.2,sd.alpha=0.02,living.rate="short",harvest.unif=TRUE, min.harvest=1000,max.harvest=2000)




**FIGURE 2:**
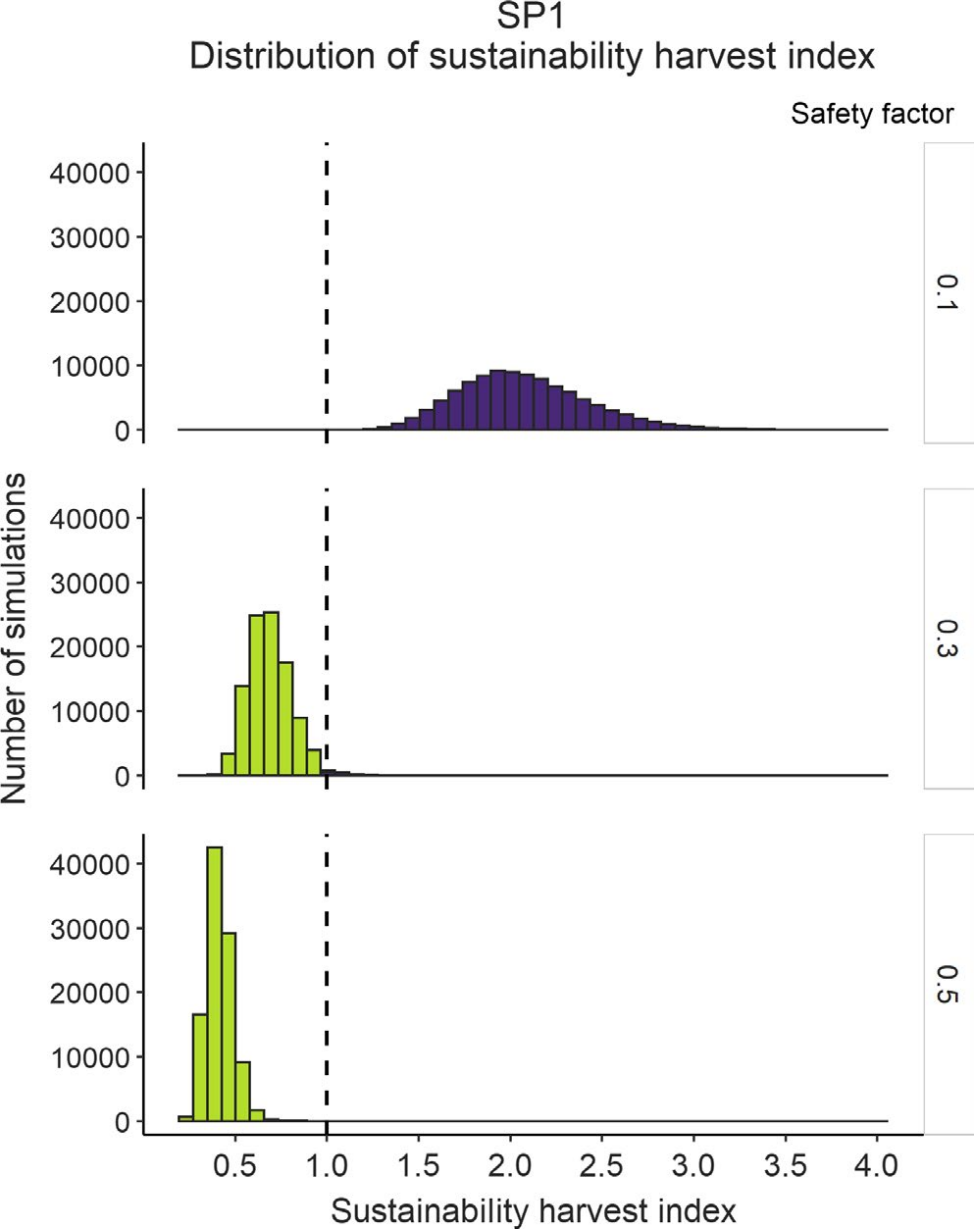
Density distribution of the simulated sustainable harvest indices under three scenarios for the safety factor. Results are from 100,000 Monte Carlo simulations performed using PEG function for a short‐lived species, including uncertainty for various initial parameters (see example “*dim*.*5*.*un*,” Appendix S1)

The output.summary (df) function provides summary statistics (mean, median, 95%CI) for relevant parameters including *r*_max_ (Rmax), potential excess growth (PEG), and the sustainable harvest index (SHI). The function also returns the percentage (with 1 = 100%) of simulations (unsustain.harvest) for which mortality level exceeds the potential excess growth (i.e., SHI > 1). The function SHI.plot(df) function allows users figuring a distribution histogram of the simulated sustainable harvest indices (SHI, Figure 2).

### 3.2 Setting allowable take levels: the PTL approach

The growth of a population exposed to additional mortality like harvest can be formulated as a theta‐logistic model, which includes density‐dependent regulation mechanisms:

(5) $$N_{t+1}=N_{t}+N_{t}\times r_{\max}\times \left[1‐{\left(\frac{N_{t}}{K}\right)}^{\theta }\right]‐H_{t}$$

*N* is the population size, *r*_max_ is the maximal annual recruitment rate, *K* is the population carrying capacity, *θ* is a shape parameter describing the shape of the density‐dependent function, and *H* is the harvest level. From this formulation, a potential and sustainable harvest level can be derived following Johnson et al. (2012):

(6) $${\text{PTL}}_{t}=F_{\text{obj}}\times \left(\frac{r_{\max}\theta }{\left(\theta +1\right)}\right)\times N_{t}$$

A critical assumption of the PTL approach is that population growth rates compensate for additional mortality through density‐dependent mechanisms (O'Brien et al., 2017). Assuming a logistic growth and a linear form of density dependence (i.e., *θ* = 1), a maximum sustainable yield (MSY) is achieved at *r*_max_ *K*/4 or at a rate *r*_max_/2. With such a rate, the population is expected to equilibrate at one‐half of its carrying capacity (*K*/2, Runge et al., 2009). If density dependence follows a concave (*θ* < 1) or a convex (*θ* > 1) form, the sustainable rate, which maximizes harvest level (MSY) is $\left(r_{\max}\theta \right)/\left(\theta +1\right)$ and the population size is expected to converge to an equilibrium point (Johnson et al., 2012) equal to:

(7) $$N_{e}=K\times {\left[1‐F_{\text{obj}}\times \frac{\theta }{\left(\theta +1\right)}\right]}^{1/\theta }$$

PTL is a useful approach to set allowable take level but also to assess whether current harvest levels are sustainable with regard to management objectives (i.e., Mortality/Potential take level <1). The PTL function provides users with an algorithm that performs these calculations on the basis of the demographic traits *N*, *r*_max_, *θ*, and current harvest levels. Architecture and syntax of this function are identical in all respects to the PEG function. Again, *r*_max_ can be approximated as *r*_max_ ≈ *λ*_max_–1 (Dillingham & Fletcher, 2008; Runge et al., 2009), so users can take advantage of all the optional ways to estimate *λ*_max_ (see Section 3.1). Theta (i.e., *θ*) is an additional argument, which describes the shape of the density dependence function. When *θ* is unknown, users may set a priori values (e.g., Koneff et al., 2017) using the arguments theta.fixed or alternatively estimate it from the equation established by Johnson et al. (2012, from Saether & Engen, 2002), relating *θ* to *r*_max_ (estim.theta):

(8) $$\ln\left(\theta \right)=1.129‐1.824\times r_{\max}+e$$

where *e* are normally distributed residuals ~*N*(0, *σ* ^2^ = 0.942). Here, users have two options: setting a deterministic relationship (*e* = 0, argument estim.theta="determinist") or taking into account errors by sampling within the distribution of residuals (argument estim.theta="random"; Table 1).



 PTL(pop.fixed=10000,NSp=1,Fobj=c(0.1,0.3,0.5),surv.fixed=0.80,alpha.fixed=1,living.rate="short",theta.fixed=1,harvest.fixed=1500)




The output.summary function returns PTL values and a *sustainable harvest index* (SHI), which assesses whether current harvest levels are sustainable with regard to management objectives. SHI is calculated as the ratio Harvest/PTL, with value >1 indicating unsustainability.

An important parameter of the PTL formulation is the scaling factor *F*_obj_, which represents management objectives. This argument is passed to the function as Fobj. It is up to the users to fix *F*_obj_ values according to the «desired long‐term population size relative to the carrying capacity» (Runge et al., 2009). *F*_obj_ can take all values along the gradient 0 < *F*_obj_ < (*θ* + 1)/*θ*. With *F*_obj_ = 1, the harvest strategy seeks achieving maximum sustained yield (MSY) and holding population size at *N*_e_ (Equation 7). With *F*_obj_ < 1, the harvest strategy is more conservative, taking only a fraction of MSY. When *F*_obj_ values approach zero, very limited harvest is allowed and population size is expected to reach its carrying capacity. At the opposite end of the gradient, when *F*_obj_ approaches (*θ* + 1)/*θ*, the harvest strategy is to hold the population at a small fraction of its carrying capacity (Johnson et al., 2012; Runge et al., 2009). Any value of *F*_obj_ produces a “sustainable” harvest strategy. Here, users should be aware that this concept has a different meaning than for the DIM approach (*cf*. 3.1). Under the PTL approach, a sustainable strategy corresponds to harvest levels that allow management objectives to be met, which does not necessarily include a reduction in the risk of population decline. Even *F*_obj_ close to (*θ* + 1)/*θ* may reflect a suitable strategy such as eradicating exotic pest species. Users should be aware that when harvest levels exceeds MSY, population equilibria are unstable. Uncontrolled variation in population size can lead either to collapse or to a growing population in the face of a constant harvest. If avoiding resource depletion is a prominent objective, *F*_obj_ should be set while taking into account important aspects, including the frequency at which population size is updated, the population carrying capacity, the strength of density dependence, the magnitude of environmental stochasticity, or the existence of additional sources of mortality (see Johnson et al., 2012; O'Brien et al., 2017).

The PTL function provides users with similar flexibility and simulation capabilities than the PEG function (Table 1, Figure 1). The output.summary() function also provides with similar summary statistics (mean, median, 95%IC) for relevant parameters including *r*_max_ (Rmax), potential take level (PTL), the sustainable harvest index (SHI), and the percentage of simulations (unsustain.harvest) for which a harvest level exceeds the potential take level. The function SHI.plot() allows users to plot the simulated sustainable harvest indices calculated under the PTL approach. Detailed examples are provided as Appendix S1.

## 4 CONCLUSION

popharvest provides wildlife managers and policymakers with a useful toolbox to assess or mitigate the impact of harvest regimes on bird populations. The package uses an intuitive programming language, allowing *R* beginners to easily perform analyses, including testing management scenarios for a set of parameters (e.g., best vs. worst scenarios) and incorporating uncertainty/stochasticity in calculations through Monte Carlo simulations. Accounting for uncertainty is a key component to limit the risk of taking wrong management decisions (Johnson et al., 2012). The propagating of uncertainty in the assessment process is one strength of the package.

In this note, both the DIM and PTL approaches are briefly described, providing users with an overview of their potential and how they are implemented in the popharvest *R* package. We refer users to the original articles in gauging the limits, advantages, and assumptions inherent to the methods covered by popharvest (e.g., Johnson et al., 2012; Niel & Lebreton, 2005; O'Brien et al., 2017; Runge et al., 2009; Wade, 1998). For instance, several important aspects and limitations are not further discussed, including the importance of having reliable and frequent estimates for both population sizes and harvest levels, the need for a relevant delineation of management units (especially for migrants), or the importance of taking into account all additional mortality sources in calculations. In the PTL approach, the continuum of possible population responses to harvest levels (compensatory–additivity/depensatory, Péron, 2013) is also overlooked, as well as some components of the underlying population dynamics (e.g., demographic stochasticity). Most often, these aspects are either imprecise or unknown in a context of limited demographic data. Accordingly, users should be aware that making some assumptions and using some key parameters/hypotheses will influence the output of the functions, with potentially important consequences on management decision. For example, using underestimated survival values that do not correspond to those observed under optimal conditions will result in overestimating growth rates and consequently the values of sustainable harvest levels. One option may be to consider a conservative approach, whether in the choice of the initial values of safety/objective factors (i.e., *Fs*, *F*_obj_), for some parameters, or in assumptions about the demographic functioning of the populations of the studied species (e.g., shape of density dependence). For instance, Wade (1998) recommends using a minimal value for population size (i.e., *N*_min_) equal to the lower bound of a 60% confidence interval (see also Dillingham & Fletcher, 2008; Runge et al., 2009). The popharvest package enables a quick and easy assessment of a multitude of scenarios including different initial values for population sizes, different shapes and strengths of density dependence, or different maximum population growth rates. Such scenarios can help inform debates about the sustainability of the exploitation of species for which little knowledge about their demography exists.

Estimates of *λ*_max_ derived from the approach of Niel and Lebreton (2005) are based on two equations, which apply differentially depending on whether the studied species is considered as long‐lived (Equation 2) or short‐lived (Equation 3). Choosing between these two categories can be tricky, especially in the context of limited demographic data, but users should be aware that this can strongly affect *λ*_max_ estimates. For the same value of alpha (*α*: age at first reproduction), *λ*_max_ calculated for a given species will be lower if the species is categorized as “long‐lived.” Simulations show that the magnitude of the difference may turn out to be very large, in particular for low values of *α*. For example, with survival = 0.7 and *α* = 3 years, *λ*_max_ will be, respectively, estimated at 1.27 and 1.23, depending on whether the species is considered as short‐ or long‐lived. More significant deviations are observed when *α* = 1 (with *s* = 0.7, *λ*_max_ = 1.87 vs. 1.55). However, with *α* = 1, a species can be categorized unambiguously as short‐lived, but uncertainty may arises for species with intermediate life‐history strategies. Niel and Lebreton (2005) do not provide explicit arguments on the threshold used to assign a species to either categories. In their original works, “short‐lived” species that warranted the use of Equation (3) were only those reproducing at 1 year (i.e., Great Tit *Parus major*, Rock Sparrow *Petronia petronia*). Thus, the use of Equation (3) only for species that breed at 1 year can be viewed as a sensible and conservative option.

We anticipate that popharvest will encourage a wider use of the DIM and PTL approaches to perform initial assessments of the consequences of harvest regimes on certain species and/or populations, and to prioritize research works or management actions. The package allows wildlife conservationists and managers to easily share scenarios (e.g., best vs. worst) and analyses using a common language that does not require an in‐depth mastery of the *R* syntax. In addition, the ease of use of the popharvest package ensures both the reproducibility of the analyses and the transparency about assumptions and initial values assigned to the various key parameters.

It is noteworthy that although popharvest focuses primarily on hunted birds, some functionalities can be used in other contexts (e.g., poaching, Brochet et al., 2016, incidental fatalities), or even for nonavian models if *λ*_max_–1 (or *r*_max_) is a known quantity.

## CONFLICT OF INTEREST

All authors declare no conflict of interest.

## AUTHOR CONTRIBUTIONS

**Cyril Eraud:** Conceptualization (equal); Methodology (equal); Software (equal); Writing‐original draft (lead). **Tiphaine Devaux:** Conceptualization (equal); Methodology (equal); Software (lead); Writing‐original draft (equal). **Alexandre Villers:** Methodology (supporting); Software (supporting); Writing‐original draft (equal). **Fred A. Johnson:** Conceptualization (equal); Methodology (equal); Software (equal); Writing‐original draft (equal). **Charlotte Francesiaz:** Conceptualization (equal); Methodology (equal); Software (supporting); Writing‐original draft (equal).

## Supporting information

Appendix S1

## Data Availability

*R* scripts and examples are provided as Appendix S1. The package (version 1.0.0; https://doi.org/10.5281/zenodo.5522675) and help files are available on Github (https://github.com/popharvest/popharvest).

## Appendix 1

### 1.1 Estimating survival from body mass

The mathematical formulation linking adult survival under ideal conditions (*S_a_*) and body mass is detailed in Johnson et al. (2012). This approach is grounded on the Slade et al. (1998)’s assumption that 1% of a new cohort survives to senescence. With *α* = age at first breeding, *ω* = age at last breeding (or maximum longevity) and *S_j_* = annual survival of all birds of age <*α* (i.e. from fledging to *α*), the original Slade et al.‘s formula takes the form:

(A1)
$$s_{a}={\left(\frac{0.01}{s_{j}}\right)}^{1/\left(\omega -\alpha \right)}$$

Johnson et al. (2012) have shown that the fraction of a cohort surviving to senescence (*p*) may achieve upper values, ranging from 0.01 to 0.08. Accordingly, they reformulated the original equation by substituting the 1% rule (i.e. *p* = .01) by the mean of their estimates (*p* = .03; SD: 0.017):

(A2)
$$s_{a}={\left(\frac{0.03}{s_{j}}\right)}^{1/\left(\omega -\alpha \right)}$$

Because *p* estimates were calculated using only data for birds aged ≥1 year (see Johnson et al., 2012), *S_j_* can be ignored when α=1. Hence:

(A3)
$$s_{a}=p^{1/\left(\omega -\alpha \right)}$$

According to Johnson et al. (2012), *p* is assumed following a beta distribution whose shape parameters (alpha = 3.34, beta = 101.24) are specified from mean and variance of *p* (*p* = .03; SD: 0.017) and the method of moments. This formulation is neither conditional on mass nor on age at sexual maturity. The link between $s_{a}$ and body mass is provided by the relationship between body mass (*M*, in kg) and maximum longevity (*ω*). In birds, Johnson et al. (2012) have shown that this relationship follows:

(A4)
$$\omega =\exp\left(3,22+0,24\times \ln(M)\right)$$

Having *p* ≈ beta (3.34, 101.24), adult survival under ideal conditions ( $s_{a}$) is estimated as:

(A5)
$$s_{a}=p^{1/\left(\exp\left[3,22+0,24\times \ln\left(M\right)\right]-\alpha \right)}$$

This formula is correct for any *α* if, and only if, $s_{j}<s_{a}$ solely for birds aged <1 year. This is a reasonable approximation especially in passerines and some other medium‐sized bird species.

If, however, $s_{j}<s_{a}$ for birds aged 1 to *α*, then the appropriate formula should revert to:

(A6)
$$s_{a}={\left(\frac{p}{s_{j}}\right)}^{1/\left(\exp\left[3,22+0,24\times \ln\left(M\right)\right]-\alpha \right)}$$

where $s_{j}$ is (average) annual survival for birds aged 1 to α (note that this contrasts to the original Slade et al.’s formula in which $s_{j}$ is annual survival from fledging to α). Unfortunately, this approach requires an estimate of $s_{j}$, which is typically very difficult to attain, even for first year birds.

By default, Equation A5 is implemented in the current version of the popharvest package (i.e. functions mass.fixed, mass.lognorm). Practically, $s_{j}$ is ignored in calculations by setting $s_{j}$ =1 in Equation A6. This means that for species with delayed sexual maturity and when $s_{j}<s_{a}$ for birds aged 1 to α, $s_{a}$ approximated from Equation A5 is negatively biased. However, if estimates of $s_{j}$ are available, users can call corresponding functions to use Equation A6 instead. This is done by passing the (fixed) argument **surv.j** = *average annual survival for birds aged 1 to* α, which is used to estimate $p=p/s_{j}$.

Using the default parametrization [Equation A5], the following example estimates survival to 0.89 and λ_max_ to ≈1.21.

PEG(full.option = TRUE, pop.fixed = 10000, NSp = 1, Fs = 0.3, mass.fixed = 2.5, type.p = "determinist", type.e = "determinist", alpha.fixed = 2, living.rate = "long", harvest.fixed = 1500).

By passing the argument **surv.j = 0.66**, the function returns 0.90 and 1.199, respectively:

PEG(full.option = TRUE, pop.fixed = 10000, NSp = 1, Fs = 0.3, mass.fixed = 2.5, **surv.j = 0.66**, type.p = "determinist", type.e = "determinist", alpha.fixed = 2, living.rate="long", harvest.fixed = 1500).
