# Supplementary material for: popharvest: An R package to assess the sustainability of harvesting regimes of bird populations
Source: Ecol Evol. 2021 Nov 15;11(23):16562–71. doi: 10.1002/ece3.8212 (PMC8668730; doi:10.1002/ece3.8212)
Supplement: Supplementary file 1 — Appendix S1 [file ECE3-11-16562-s001.pdf]

Supporting Information:

popharvest : An *R* package to assess the *sustainability of harvesting* regimes of bird populations

Cyril Eraud[1], Tiphaine Devaux[1,2], Alexandre Villers[1], Fred A. Johnson[3] & Charlotte Francesiaz[4]

[1] Office Français de la Biodiversité, Direction de la Recherche et de l'Appui Scientifique, Unité Avifaune migratrice, 79360 Villiers-en-Bois, France

[2] Office de Génie Écologique, Saint-Maur-Des-Fossés, France

[3] Aarhus University, Department of Bioscience – Kalø, DK-8410 Rønde, Denmark

[4] Office Français de la Biodiversité, Direction de la Recherche et de l'Appui Scientifique, Unité Avifaune migratrice, 85340 Ile-D'Olonne, France

Correspondence: Cyril Eraud. E-mail : cyril.eraud@ofb.gouv.fr

```r
# popharvest : An R package to assess the sustainability of harvesting regimes
# of bird populations
# Cyril Eraud(1), Tiphaine Devaux, Alexandre Villers, Fred A. Johnson & Charlotte Francesiaz
# (1) cyril.eraud@ofb.gouv.fr

# Installing package from Github
library(devtools)
install_github("popharvest/popharvest")
library(popharvest)
```

Code and examples for the DIM approach (detecting overharvest)

```r
##################################################################################
#
# Code and examples for the DIM approach (detecting overharvest)
##################################################################################

#### dim.1
# SIMPLE EXAMPLE WITH LAMBDA MAX #
# Example 1: Suppose a population of 10 000 individuals with an annual maximal
# growth rate of 1.35, and a take level of 1 500 individuals. A "safety factor" is
# set at 0.3. There is only one studied population (NSp=1).

dim.1<-PEG(pop.fixed=10000, NSp= 1, Fs=0.3, lambdaMax.fixed=1.35, harvest.fixed =1500)
dim.1
##   SP Sim  Fs Rmax popsize harvest  PEG      SHI
## 1  1   1 0.3 0.35   10000    1500 1050 1.428571

# Return a dataframe with 8 variables:
# $ SP     : ID of the species/population
# $ Sim  : index of the simulation (default value is IDsim = 1)
# $ Fs     : value of the safety factor
# $ Rmax   : value of the maximal annual recruitment rate (lambda max - 1)
# $ popsize: population size (in number of individuals)
# $ harvest: number of individuals harvested each year
# $ PEG : calculated value of the Potential excess growth
# $ SHI : calculated value of the Sustainable hunting index (if>1, harvest exceeds PEG)

#### dim.2
# ADD SEVERAL LEVELS FOR THE SAFETY FACTOR #
# Example 2: Similar analysis as in example 1, but with 3 values for the
#"safety factor" (0.1, 0.2, 0.3)

dim.2<-PEG(pop.fixed=10000, NSp= 1, Fs=c(0.1, 0.2, 0.3), lambdaMax.fixed=1.35, harvest.fixed
=1500)
dim.2
##   SP Sim  Fs Rmax popsize harvest  PEG      SHI
## 1  1   1 0.1 0.35   10000    1500  350 4.285714
## 2  1   1 0.2 0.35   10000    1500  700 2.142857
## 3  1   1 0.3 0.35   10000    1500 1050 1.428571



#### dim.3
# STUDY SEVERAL SPECIES IN THE SAME RUN #
# Example 3: Similar analysis as in former examples, but for 2 species (or
# populations)in the same run. Population size is respectively 10 000 and
# 20 000. Lambda max is 1.35 for the first pop/species, 1.10 for the second.
# Take levels are respectively: 1 500 and 300 individuals each year.
```

```
dim.3<-PEG(pop.fixed=c(10000, 20000), NSp= 2, Fs=c(0.1,0.2,0.3), lambdaMax.fixed=c(1.35, 1.10),
harvest.fixed = c(1500, 300))
dim.3
##   SP Sim  Fs Rmax popsize harvest  PEG      SHI
## 1  1   1 0.1 0.35   10000    1500  350 4.285714
## 2  1   1 0.2 0.35   10000    1500  700 2.142857
## 3  1   1 0.3 0.35   10000    1500 1050 1.428571
## 4  2   1 0.1 0.10   20000     300  200 1.500000
## 5  2   1 0.2 0.10   20000     300  400 0.750000
## 6  2   1 0.3 0.10   20000     300  600 0.500000

#### dim.4
# SIMPLE CASE WITH DEMOGRAPHIC PARAMETERS #
# Example 4: Similar analysis as example 1, but lambda max is unknown and estimated
# from adult survival (surv.fixed = 0.8) and age at first breeding (alpha.fixed = 1).
# The species has a short life-history strategy (living.rate=short).

dim.4<-PEG(pop.fixed=10000, NSp= 1, Fs=0.3, surv.fixed=0.80, alpha.fixed=1,
living.rate="short", harvest.fixed = 1500)
dim.4
##   SP Sim  Fs      Rmax popsize harvest      PEG       SHI
## 1  1   1 0.3 0.6960904   10000    1500 2088.271 0.7182975
# From survival and alpha, Rmax is estimated to 0.696.
# PEG and SHI are calculated accordingly.

#### dim.5
# SIMPLE CASE WITH SURVIVAL ESTIMATED FROM ALLOMETRIC RELATIONSHIP #
# Example 5: Similar analysis as in example 4 but Lambda max (or Rmax) and survival are both
unknown.
# Age at first breeding is 1 year. The species has a short life-history strategy.
# Survival is estimated from body mass (0.125 kg; mass.fixed=0.125), assuming p and residuals
(e) at their means.
# See Eq. 4 in article.

dim.5<-PEG(pop.fixed=10000, NSp= 1, Fs=0.3, mass.fixed=0.125, type.p = "determinist", type.e =
"determinist", alpha.fixed=1, living.rate="short", harvest.fixed = 1500)
dim.5
##   SP Sim  Fs      Rmax popsize harvest      PEG       SHI
## 1  1   1 0.3 0.7248509   10000    1500 2174.553 0.6897971
# By passing the argument full.option=TRUE returns all values used to estimate PEG and SHI,
including p and e.

dim.5.fo<-PEG(full.option=TRUE, pop.fixed=10000, NSp= 1, Fs=0.3, mass.fixed=0.125, type.p =
"determinist", type.e = "determinist", alpha.fixed=1, living.rate="short", harvest.fixed =
1500)
dim.5.fo
##   SP Sim  Fs      Rmax living.rate lambdaMax  survival  mass e          p
## 1  1   1 0.3 0.7248509       short  1.724851 0.7845635 0.125 0 0.03193727
##   surv.j alpha popsize harvest      PEG       SHI
## 1      1     1   10000    1500 2174.553 0.6897971
# From body mass, survival is estimated to 0.785, then Rmax to 0.725.
# PEG and SHI are calculated accordingly.

# Another option with uncertainty in p and e.
# 100 000 simulations.

set.seed(1234)
dim.5.un<-PEG(full.option=TRUE, Nsim=100000, pop.fixed=10000, NSp= 1, Fs=c(0.1, 0.3, 0.5),
mass.fixed=0.125, type.p = "random", type.e = "random", alpha.fixed=1, living.rate="short",
harvest.fixed = 1500)
```

```
round(output.summary(dim.5.un), 3) # Rounded values
##                         SP1      SP1      SP1
## Fs                    0.100    0.300    0.500
## med.Rmax              0.735    0.736    0.735
## mean.Rmax             0.744    0.745    0.745
## percent2.5.Rmax       0.525    0.525    0.525
## percent97.5.Rmax      1.014    1.019    1.018
## med.PEG             735.000 2209.000 3676.000
## mean.PEG            744.000 2236.000 3724.000
## percent2.5.PEG      525.000 1576.000 2626.000
## percent97.5.PEG    1014.000 3056.000 5089.000
## med.SHI               2.041    0.679    0.408
## mean.SHI              2.075    0.690    0.414
## percent2.5.SHI        1.480    0.491    0.295
## percent97.5.SHI       2.857    0.952    0.571
## unsustain.harvest     1.000    0.012    0.000
mean(dim.5.un$survival)
## [1] 0.7691519
mean(dim.5.un$Rmax)
## [1] 0.7446553

# Here mean survival is estimated to 0.77, then mean Rmax to 0.74.
# With Fs=0.1, mean PEG is 744 and mean SHI is 2.075.

SHI.plot(dim.5.un, color1="#B4DE2CFF", color2 = "#482878FF")
```

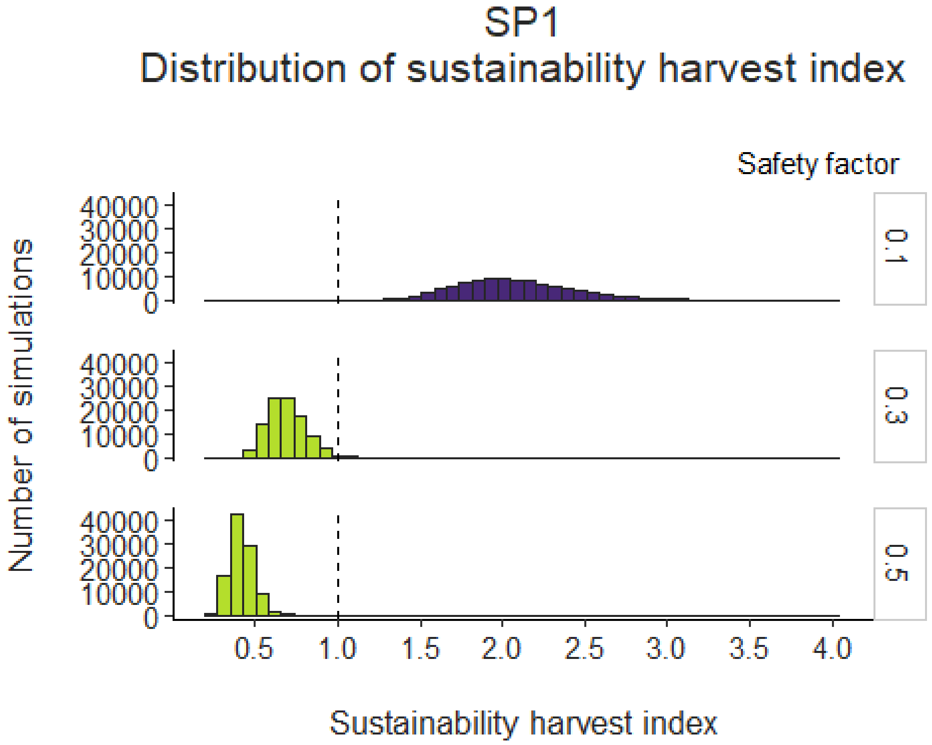


```
# dim.5.un$unsustain.harvest gives the % (with 1 = 100%) of simulations with SHI value>1.
# Here, with Fs=0.1, all simulations (100%) are >1 suggesting overharvest.
# With Fs=0.3, the percentage is 1.2%.

#### dim.6
# ADD UNCERTAINTY FOR ALL PARAMETERS #
# Example 6: One population. The species has a short life-history strategy.
# lamda max is unknown and estimated from survival and age at first reproduction.
```

```
# Uncertainty for the following parameters: population size follows a uniform distribution
# U(10000,20000), survival follows a beta distribution Beta(a, B) with a and B estimated from
mean (0.65) and standard deviation (0.08),
# age at first reproduction is log-normally  distributed as lnN(1.2, 0.02),
# and harvest level is estimated as ranging from 1 000 and 2 000 individuals (i.e.
U(1000,2000)).
# The safety factor is set to 0.3.
# 100 000 Monte-Carlo simulations are performed.

set.seed(1234)
dim.6<-PEG(Nsim=100000, NSp= 1, Fs=0.3, pop.unif=TRUE, min.pop=10000,
           max.pop=20000, surv.beta =TRUE, mean.surv=0.65, sd.surv=0.08,
           alpha.lognorm=TRUE, mean.alpha=1.2, sd.alpha=0.02, living.rate="short",
           harvest.unif=TRUE, min.harvest=1000, max.harvest=2000)

results.dim.6<-output.summary(dim.6)
##                          SP1
## Fs                     0.300
## med.Rmax               0.744
## mean.Rmax              0.743
## percent2.5.Rmax        0.562
## percent97.5.Rmax       0.920
## med.PEG             3291.000
## mean.PEG            3340.000
## percent2.5.PEG      2051.000
## percent97.5.PEG     4879.000
## med.SHI                0.452
## mean.SHI               0.475
## percent2.5.SHI         0.252
## percent97.5.SHI        0.818
## unsustain.harvest      0.003

SHI.plot(dim.6, color1 = "#B4DE2CFF", color2 = "#482878FF")
```

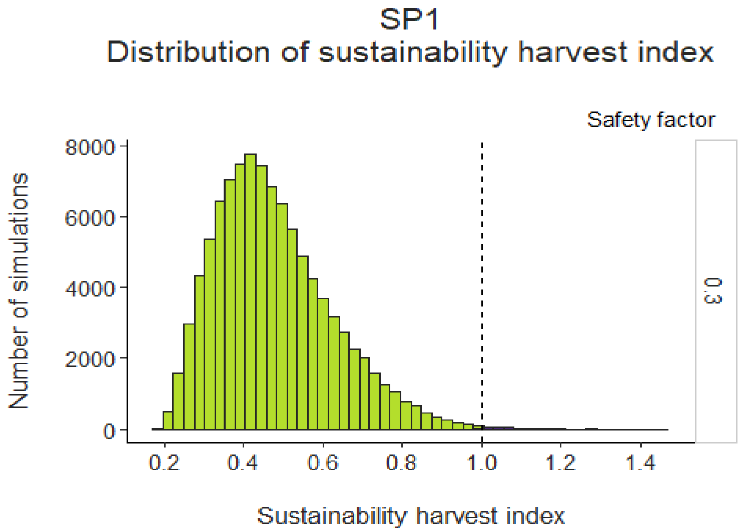


```
# UNCERTAINTY, 2 SPECIES, 2 LEVELS OF SAFETY FACTORS
# Example 7

#### dim.7
# Example 7: 2 species (or populations) with a short life-history strategy.
# lamda max is unknown and estimated from survival and age at first reproduction.
# Uncertainty for the following parameters: population size follows a uniform distribution
# U(10000,20000) for the first species and U(30000, 60000) for the second. Survival follows
```

```r
# a beta distribution Beta(a, B) with a and B estimated from mean (0.65 and 0.75)
# and standard deviations (0.06 and 0.07, respectively).
# Age at first reproduction is one year for both species.
# Harvest level is estimated to 1 000 - 2 000 individuals for the first species, 500 - 1 000
# for the second.
# The safety factor is set to 0.3 and 0.01 for both species.
# 100 000 Monte-Carlo simulations are performed.

set.seed(1234)
dim.7<-PEG(full.option=TRUE, Nsim=100000, NSp= 2, Fs=c(0.1, 0.3), pop.unif=TRUE,
min.pop=c(10000, 30000),
           max.pop=c(20000,60000), surv.beta =TRUE, mean.surv=c(0.65, 0.75),
sd.surv=c(0.06,0.07),
           alpha.fixed = c(1,1), living.rate=c("short","short"),
           harvest.unif=TRUE, min.harvest=c(1000, 500), max.harvest=c(2000,1000))

results.dim.7<-output.summary(dim.7)
##                        SP1      SP1      SP2       SP2
## Fs                   0.100    0.300    0.100     0.300
## med.Rmax             0.945    0.944    0.778     0.779
## mean.Rmax            0.944    0.943    0.779     0.779
## percent2.5.Rmax      0.764    0.764    0.544     0.544
## percent97.5.Rmax     1.118    1.119    1.018     1.019
## med.PEG           1402.000 4207.000 3437.000 10304.000
## mean.PEG          1415.000 4246.000 3509.000 10510.000
## percent2.5.PEG     904.000 2710.000 2041.000  6098.000
## percent97.5.PEG   2005.000 6019.000 5337.000 15991.000
## med.SHI              1.064    0.354    0.216     0.072
## mean.SHI             1.112    0.371    0.228     0.076
## percent2.5.SHI       0.606    0.202    0.117     0.039
## percent97.5.SHI      1.880    0.627    0.406     0.136
## unsustain.harvest    0.581    0.000    0.000     0.000

SHI.plot(dim.7, NameSp = c("Species A", "Species B"), color1 = "#B4DE2CFF", color2 =
"#482878FF")
```

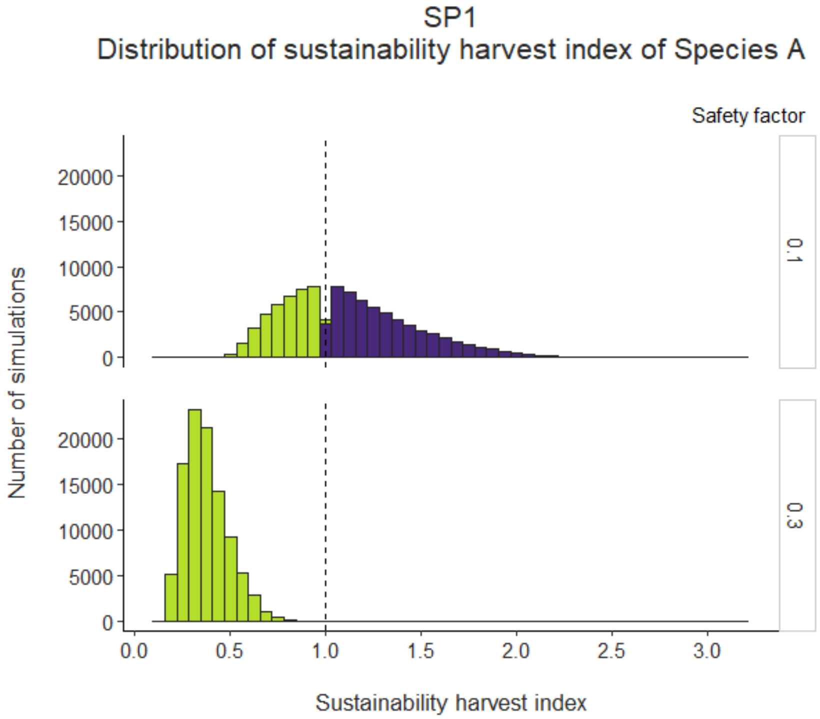

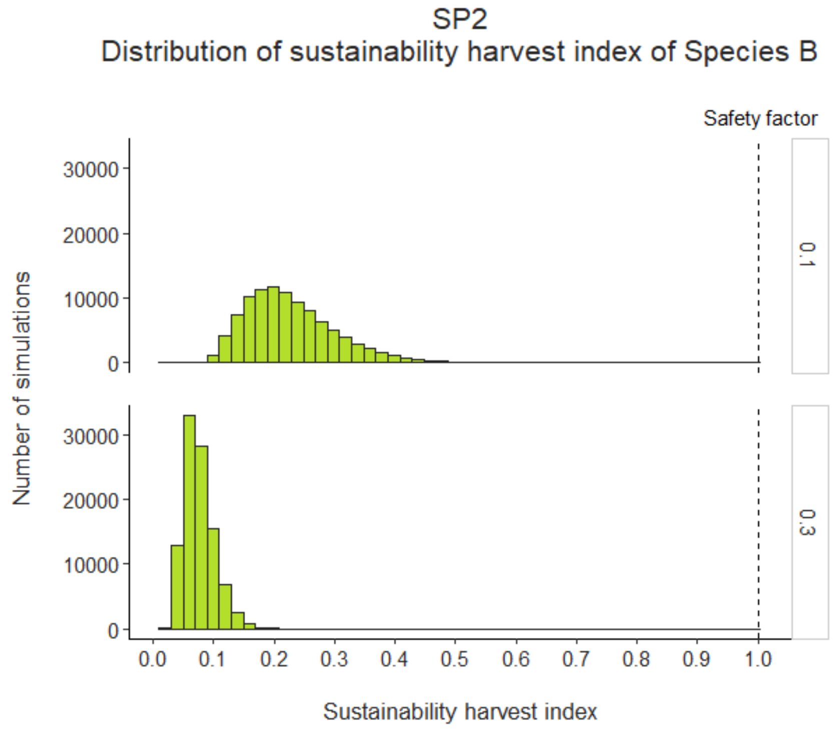


```
# The function returns one figure per species
# Each figure shows the results for each of the 2 levels of safety factor
```

Code and examples for the PTL approach (setting allowable take levels)

```
################################################################################
#
# Code and examples for the PTL approach (setting allowable take levels)
################################################################################

#### ptl.1
# SIMPLE EXAMPLE WITH FIXED SURVIVAL #
# Example 1: Suppose a population of 10 000 individuals (pop.fixed=10000) with a
# survival rate of 0.80 (surv.fixed=0.80), and a take level of 1 500 individuals per year
(harvest.fixed=1500).
# There is only one studied population (NSp=1).
# Management objective is set at 0.5 (Fobj=0.5).

ptl.1<-PTL(pop.fixed=10000, NSp=1, Fobj=0.5, surv.fixed = 0.80, alpha.fixed = 1, living.rate=
"short", theta.fixed=1, harvest.fixed = 1500)
ptl.1
##   SP Sim Fobj      Rmax popsize theta harvest      PTL      SHI
## 1  1   1  0.5 0.6960904   10000     1    1500 1740.226 0.861957

#### ptl.2
# SIMPLE EXAMPLE WITH FIXED SURVIVAL BUT SEVERAL MANAGEMENT OBJECTIVES #
# Example 2
ptl.2<-PTL(pop.fixed=10000, NSp=1, Fobj=c(0.1,0.3,0.5), surv.fixed = 0.80, alpha.fixed = 1,
living.rate= "short", theta.fixed=1, harvest.fixed = 1500)
ptl.2
```

```
##   SP Sim Fobj      Rmax popsize theta harvest       PTL      SHI
## 1  1   1  0.1 0.6960904   10000     1    1500  348.0452 4.309785
## 2  1   1  0.3 0.6960904   10000     1    1500 1044.1356 1.436595
## 3  1   1  0.5 0.6960904   10000     1    1500 1740.2260 0.861957

#### ptl.3
# SIMPLE EXAMPLE WITH SURVIVAL ESTIMATED FROM BODY MASS #
# Example 3: Suppose a population of 10 000 individuals (pop.fixed=10000) with a survial
# estimated from the species's mass (0.125kg, mass.fixed=0.125).
# The take level is 1 500 individuals per year (harvest.fixed=1500).
# One studied population (NSp=1).

ptl.3<-PTL(pop.fixed=10000, NSp= 1, Fobj=0.3, mass.fixed=0.125, type.p = "determinist", type.e
= "determinist", alpha.fixed=1, living.rate="short", theta.fixed = 1,  harvest.fixed = 1500)
ptl.3
##   SP Sim Fobj      Rmax popsize theta harvest      PTL      SHI
## 1  1   1  0.3 0.7248509   10000     1    1500 1087.276 1.379594

#### ptl.4
# SEVERAL SPECIES IN A RUN #
# Example 4: Similar analysis as in former examples, but for 2 species (or populations)
# and 3 management objectives in the same run.
# Population size is respectively 10 000 and 20 000. Survival is 0.8 and 0.6.
# Age at first breeding is 1 year.
# Annual take levels are 1 500 and 300 individuals, respectively.
# The shape for density dependence is assumed as linear for both species (theta.fixed=1)

ptl.4<-PTL(pop.fixed=c(10000, 20000), NSp=2, Fobj=c(0.1,0.3,0.5), surv.fixed = c(0.80, 0.60),
alpha.fixed = c(1,1), living.rate= c("short", "short"), theta.fixed=c(1,1), harvest.fixed =
c(1500,2800))
ptl.4
##   SP Sim Fobj      Rmax popsize theta harvest       PTL       SHI
## 1  1   1  0.1 0.6960904   10000     1    1500  348.0452 4.3097850
## 2  1   1  0.3 0.6960904   10000     1    1500 1044.1356 1.4365950
## 3  1   1  0.5 0.6960904   10000     1    1500 1740.2260 0.8619570
## 4  2   1  0.1 1.0196253   20000     1    2800 1019.6253 2.7461068
## 5  2   1  0.3 1.0196253   20000     1    2800 3058.8759 0.9153689
## 6  2   1  0.5 1.0196253   20000     1    2800 5098.1265 0.5492214

#### ptl.5

# THE SHAPE OF DENSITY-DEPENDENCE (THETA) IS UNKNOWN
# Example 5: Two populations with different populations sizes (sp1= 360 000, sp2= 4 000 000),
# different life-history strategies (long vs. short lived), different (uncertain) survival
# rates (0.8, 0.65) and different (fixed) ages at first breeding (2 vs. 1 years).
# The shape of density dependence (theta) is unknown and estimated from the equation relating
# theta to Rmax.
# No error is assumed in theta estimate (residuals are set at their mean=0): "determinist".
# Annual harvest levels are 15 000 and 400 000, respectively.
# 100 000 Monte-Carlo simulations are performed, for 2 management objectives (0.3, 0.5).

set.seed(1234)
ptl.5<-PTL(full.option=TRUE, Nsim = 100000, NSp = 2, living.rate = c("long", "short"),
           surv.beta =TRUE, mean.surv=c(0.8, 0.65), sd.surv=c(0.04, 0.08),
           alpha.fixed = c(2,1), pop.fixed = c(360000, 4000000),
           estim.theta = c("determinist", "determinist"), harvest.fixed = c(15000, 400000),
           Fobj = c(0.3, 0.5))

results.ptl.5<-output.summary(ptl.5)
##                           SP1       SP1        SP2        SP2
## Fobj                    0.300     0.500      0.300      0.500
```

```
## med.Rmax               0.269     0.269      0.941      0.943
## mean.Rmax              0.269     0.269      0.940      0.941
## percent2.5.Rmax        0.223     0.223      0.701      0.699
## percent97.5.Rmax       0.312     0.313      1.174      1.173
## med.PTL            19020.000 31681.000 401187.000 668566.000
## mean.PTL           18968.000 31612.000 397838.000 662996.000
## percent2.5.PTL     16200.000 27027.000 373519.000 622551.000
## percent97.5.PTL    21465.000 35789.000 404631.000 674383.000
## med.SHI                0.789     0.473      0.997      0.598
## mean.SHI               0.795     0.477      1.006      0.604
## percent2.5.SHI         0.699     0.419      0.989      0.593
## percent97.5.SHI        0.926     0.555      1.071      0.643
## unsustain.harvest      0.003     0.000      0.433      0.000

SHI.plot(ptl.5, NameSp = c("Species A", "Species B"), color1 = "#B4DE2CFF", color2 =
"#482878FF")
```

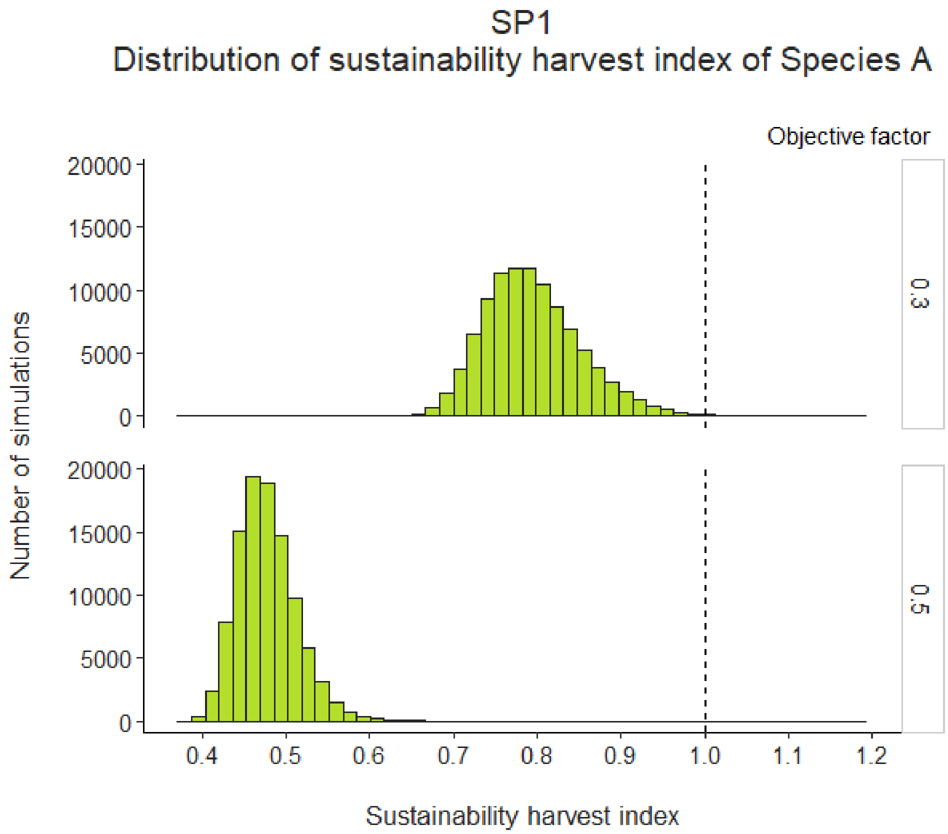
SP1
Distribution of sustainability harvest index of Species A
Objective factor
0.3
0.5
Number of simulations
0
5000
10000
15000
20000
0.4
0.5
0.6
0.7
0.8
0.9
1.0
1.1
1.2
Sustainability harvest index

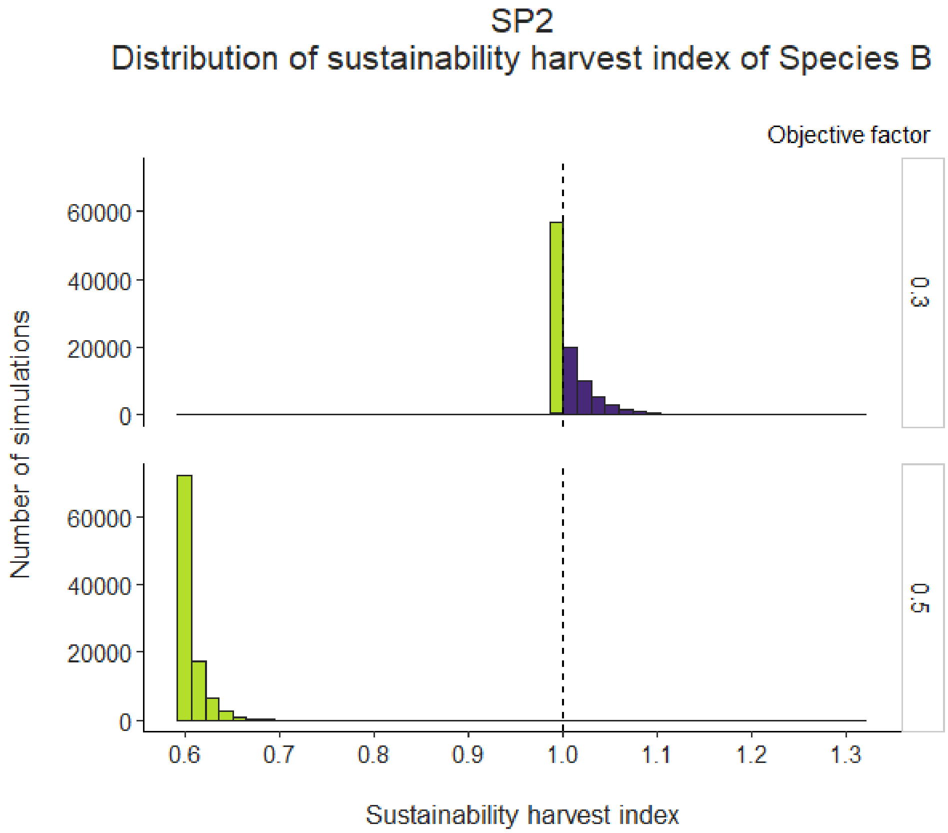
SP2
Distribution of sustainability harvest index of Species B
Objective factor
0.3
0.5
Number of simulations
0
20000
40000
60000
0.6
0.7
0.8
0.9
1.0
1.1
1.2
1.3
Sustainability harvest index
